# LEARNS Model as Perioperative Education Strategy for Patients with Laryngeal Tumors

**DOI:** 10.1155/2022/3360966

**Published:** 2022-10-12

**Authors:** Biaoxin Zhang, Qinzhi Sun, Shaohua Hu, Yinxiang Yu, Cuixia Hu, Dejuan Zhao, Jing Xu, Jun Fang, Lulu Wang

**Affiliations:** ^1^Department of Otolaryngology Head and Neck Surgery, The First Affiliated Hospital of Anhui Medical University, Hefei, Anhui 230022, China; ^2^Nursing Department, The First Affiliated Hospital of Anhui Medical University, Hefei, Anhui 230022, China

## Abstract

**Objective:**

To evaluate LEARNS model as a perioperative strategy for health education and nursing supervision of patients with laryngeal tumors.

**Methods:**

LEARNS scheme based on the best practice guidelines was applied to patients in the observation group: (1) analyze the needs of patients (Listen_L); (2) establish therapeutic partnership (Establish_E); (3) adopt intentional intervention (Adopt_A); (4) reinforce health awareness (Reinforce_R); (5) implement feedback assessment of knowledge (Name_N); (6) strengthen self-management based on community resources (Strengthen_S). In the control group, traditional medical care instructions were provided to the patients by medical staff. Parameters such as anxiety status, treatment compliance, nursing satisfaction, self-care ability, and life quality were compared between the observation and control groups.

**Results:**

Upon admission, there was no significant difference in self-care ability and anxiety level between two groups. However, the anxiety level of observation group was significantly lower than that of the control group 1 day before operation and 7 days after operation. Postoperative treatment compliance and nursing satisfaction were also improved in the observation group. In addition, self-care ability and life quality in the observation group were significantly enhanced as compared to the control group.

**Conclusion:**

As a mutual learning process between nurses and patients, LEARNS model motivates nurses to assess the needs of patients voluntarily. Furthermore, evidence-based education reinforces the self-care ability and health awareness of the patients. Our data suggests that LEARNS model is of great value in improving the life quality of the patients with laryngeal tumors and nursing satisfaction.

## 1. Introduction

Larynx is an important organ for breathing, swallowing, and pronunciation. Laryngectomy is the main treatment for laryngeal tumors. Due to the complete or partial loss of laryngeal function after laryngectomy, patients need to wear a tracheal tube or fistula for a long time as a medical assistance [1]. After the operation, patients will suffer from a series of changes in physical, psychological and social life, including impaired breathing and swallowing capability [2], functional aphonia [3], depression and anxiety [4], uncertainty feeling of illness [5], malnutrition [6], interpersonal disorders and impaired life quality [7]. In the meanwhile, patients need to master a series of self-care skills related to wound care, airway management, diet and nutrition, and other self-care management knowledge after radiotherapy and chemotherapy [8]. Therefore, postoperative orientation for patients with laryngeal tumors is of great value to enhance self-management ability and prepare for postoperative challenges.

The LEARNS model based on best practice guidelines is a mature educational module for patient-centered and patient-oriented health awareness promotion [9]. LEARNS model encourage nurses to actively converse with patients instead of waiting for their complaints. The LEARNS processes requires nurses to actively assess the needs of patients (Listen), establish a beneficial therapeutic partnership (Establish), adopt intentional intervention methods (Adopt), reinforce health awareness of the patients (Reinforce), implement feedback assessment of health literacy (Name), and strengthen self-management strategies (Strengthen) [10]. The core guideline is to create a client-centered care environment without embarrassment and blame. Nurses serve as catalysts to start conversations with patients to promote their health awareness, build knowledge and skills essential for postoperative care and formulate self-management strategies. The final goal is to enhance health literacy and self-efficacy of the patients, which has been proved to optimize patient outcomes and self-management in different scenarios [10–12]. However, this type of patient-centered orientation program is seldom adopted in the health care system of Chinese hospitals.

In this study, we aimed to investigate the effect of LEARNS model on health education and nursing supervision of patients with laryngeal tumors. Among 60 patients who were diagnosed with laryngeal cancer, 30 patients in the control group were provided with the regular health education and 30 patients in the experimental group (observation group) were trained with the LEARNS health education model. Perioperative anxiety level, patients' compliance during the treatment, satisfaction degree of nursing staff, perioperative self-care ability and the quality of life between the two groups of patients were assessed. Based on our study, a standardized procedure of perioperative health education for patients with laryngeal tumors could improve the health literacy, self-management ability and life quality of patients with laryngeal tumors.

## 2. Methods

### 2.1. Subjects Enrollment

A total number of 60 patients who were diagnosed with laryngeal cancer and underwent laryngeal tumor resection in the Otorhinolaryngology Head and Neck Surgery Department of our hospital in Anhui Province from January 2019 to April 2021 were enrolled in this study. This study was approved by the Ethics Committee of The First Affiliated Hospital of Anhui Medical University. All the 60 patients signed the written informed consent.

#### 2.1.1. Inclusion Criteria

(1) Patients diagnosed pathologically with laryngeal tumors and underwent laryngectomy; (2) patients were in a stable condition and able to cooperate with the guidelines and instruction given by the nurses; (3) patients were capable of listening, speaking, reading and writing, with the age ≥18 years old; (4) patients were recruited on the voluntary basis and signed the informed consent.

#### 2.1.2. Exclusion Criteria

(1) Patients diagnosed with the recurrence or metastasis of laryngeal tumors in the past six months; (2) patients diagnosed with other type of tumors or mental illnesses in the treatment stage; (3) patients suffered from severe heart, lung, liver and kidney dysfunction; (4) patients suffered from severe communication disorders or unable to correctly understand the questionnaire and instructions provided.

#### 2.1.3. Falling-Off Criteria

Patients with incomplete data and those who did not complete the survey and questionnaires for various reasons.

### 2.2. Grouping Method

According to the order of admission time, enrolled subjects were randomly divided into two groups. 30 patients in the control group were provided with the regular health education model, and 30 patients in the experimental group (observation group) were trained with the LEARNS health education model. Fisher randomization technique was employed for random assignment. The statistical power of sample size was evaluated using online tool: https://www.stat.ubc.ca/∼rollin/stats/ssize/n2.html.

### 2.3. Intervention Measures for the Control Group (Conventional Health Education Model)

Perioperative health education was provided in accordance with the general nursing routines and health education pathways of surgical patients, including preoperative examination, preoperative preparation, psychological care, postoperative body movement, diet guidance, postoperative tracheal tube care (such as airway humidification, Sputum suction, cannula disinfection, pronunciation and communication method guidance), medication guidance, discharge guidance and return visit education.

### 2.4. Experimental Group (LEARNS Model) Intervention Measures

#### 2.4.1. Establishment of an Evidence-Based Nursing Research Team

An evidence-based nursing research team comprising head nurses, nursing backbones, and guideline inspectors in the department was formed to implement the LEARNS model. The research team was divided into three subgroups: nursing management team, clinical practice team, and quality control team. Members of each group summarized and discussed the research process and problems on a monthly basis.

#### 2.4.2. Conduct Evidence-Based Nursing Training on the Research Team

Domestic experts on evidence-based nursing was invited to conduct homogenization training for research team members on evidence-based medicine, evidence-based nursing and application of best practice guidelines to ensure the standardized conduct of interventions.

#### 2.4.3. Gap Analysis

To analyze the gap between the best practice guidelines based on LEARNS model and the current status of perioperative health education for patients with laryngeal tumors (see Table 1 for details).

#### 2.4.4. Assessment of the Learning Needs

The learning needs assessment questionnaires were distributed to patients in the observation group on a regular basis. The content included: study time, ways of learning, study tools, study content, and available resources. A descriptive analysis of the evaluation results was shown in Table 2.

#### 2.4.5. Clinical Application of LEARNS Model

**Figure 1:**
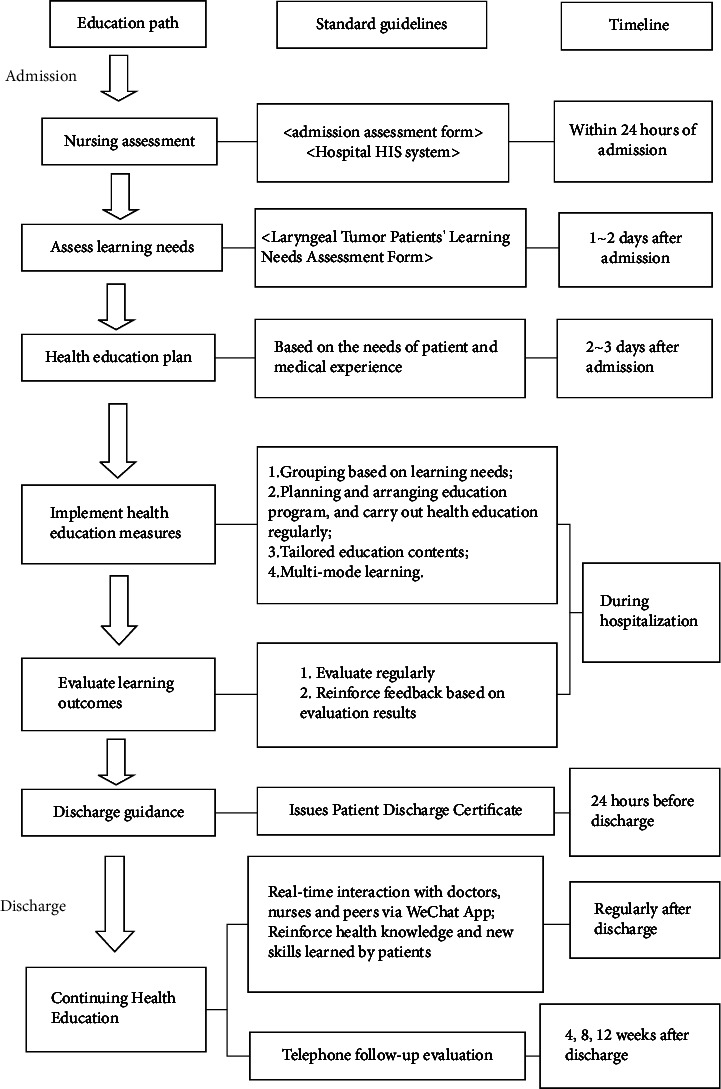
Health education flow chart of learns mode for patients with laryngeal tumors during perioperative period.

According to the LEARNS model, we identified the existing problems in the current perioperative health education for patients with laryngeal tumors and assessed the learning needs of the patients. We reformulated the process of perioperative health education for patients with laryngeal tumors, as shown in Figure 1.

### 2.5. Observation Index

#### 2.5.1. General Information Questionnaire

Designed by the research team and contained surveys about general sociodemographic data, clinical nursing data and health education status. General sociodemographic information includes: gender, age, occupation, habitual residence address, marital status, religious belief, contact information, education level, economic level, and method of payment of medical expenses. Clinical nursing information includes: operation time, operation method, tracheal cannula type, airway humidification method, presence or absence of difficulty in expectoration, cannula blockage, bleeding, decoupling, subcutaneous emphysema and other complications. The status of health education includes: concerns, degree of coping with distress, health education obtained, knowledge requirements and specific content, family and social support.

#### 2.5.2. Anxiety Self-Rating Scale (Self-Rating Anxiety Scale, SAS)

Containing 20 items (Supplementary file), each item was based on a 4-point scoring method (from 1 to 4 points). The total score was calculated by summing up the scores of all items. The total rough score was multiplied by 1.25 and the integer part was considered as the standard score. The full score was set as 100 points. The critical cutoff value for SAS was 50 points. Higher total score indicated a higher level of anxiety. Evaluations were performed on admission, 1 day before surgery and 7 days after surgery.

#### 2.5.3. Evaluation of Treatment Compliance

Score A: actively accepted the treatment plan and nursing guidance, and successfully completed the treatment. Score B: partially accepted treatment plan and nursing guidance, and completed most of the treatment and nursing care procedures. Score C: those who did not understand the treatment plan and occasionally did not cooperate with the treatment and nursing care. Evaluation was performed on the 7th day after surgery.

#### 2.5.4. Patient and Family Satisfaction

Self-designed nursing satisfaction questionnaire; the total score was 100 points; patients with ≥96 points were considered as very satisfied, patients with 90–95 points were relatively satisfactory, and those with <90 points were unsatisfactory. The assessment was conducted on the day of discharge.

#### 2.5.5. Self-Care Ability Scale (Exercise Self-Care Agency, ESCA)

Composed of 4 dimensions of self-care skills (12 items), self-responsibility (8 items), self-management literacy (9 items) and health knowledge level (14 items), with a total of 43 items (Supplementary file). A score of 0 to 4 (Five-level scoring method) was assigned to each item, with a total score of 172 points. 0 = very different from me, 1 = something not like me, 2 = no comment, 3 = something like me, 4 = very like me.

According to the total score of the scale and the score of each dimension, the self-care abilities were divided into 3 levels: 0–57 as low level; 58–115 as medium level; 116–172 as high level. A higher score of each dimension indicated a stronger self-care ability. The CVI (Core Values Index) value for the validity of the scale was 0.98, and the Cronbach's coefficient was 0.93. The evaluation was performed 4 weeks after the operation.

#### 2.5.6. Washington Medical University Quality of Life Scale (The University of Washington Quality of Life Scale, UW-QOL)

UW-QoL is one of the most frequently reported health-related quality of life (HR-QoL) questionnaires specifically in head and neck cancer, and since its first publication in 1993, it has been used in many different cohorts [13, 14]. This self-assessment questionnaire was dedicated to patients with head and neck tumors including 12 specific questions concerning the quality of life, 3 comprehensive questions༈Patients can add questions that are not included in the scale༉. The 12 specific items were: pain, appearance, entertainment, vitality, swallowing, chewing, language, shoulder function, taste, saliva, mood and anxiety. Each item was divided into 3 to 5 levels, and the score ranged from 0 to 100 points. The higher the score, the better the quality of life is. The assessment was carried out 12 weeks after the operation.

### 2.6. Data Processing and Statistical Analysis

Epidata3.1 software was used for data entry and SPSS18.0 software was employed for statistical analysis. Measurement data were described by (mean ± SD : *X* ± *S*); *t*-test was used for comparison between two groups in anxiety score before and after surgery; count data were described by case; Chi-square (*χ*^2^) statistic test and rank sum test were used for comparison of parameters including, treatment compliance, nursing satisfaction, self-management ability and quality of life between two groups. The test level *a* = 0.05, and the difference was considered as statistically significant when *P* < 0.05.

## 3. Results

### 3.1. Anxiety Level Comparison

We first assessed the anxiety level of the patients enrolled in the control group and experimental group. There was no significant difference in anxiety between the two groups of patients when they were admitted to the hospital; the anxiety level of the observation group was significantly lower than that of the control group 1 day before surgery and 7 days after surgery (Table 3).

### 3.2. Treatment Compliance and Nursing Satisfaction Comparison

The treatment compliance of the two groups of patients was evaluated on the 7th day after operation, and the nursing satisfaction was evaluated on the day of discharge. Our data demonstrated that the observation group with LEARNS model showed a better treatment compliance and a relatively higher level of nursing satisfaction (Table 4).

### 3.3. Comparison of Self-Care Ability and Quality of Life

The self-care ability of patients was evaluated at 4 weeks after operation, and the quality of life was measured at 12 weeks after operation. The observation group with the implementation of LEARNS model showed a significantly higher level of self-care capacity and life quality than the control group (Table 5).

## 4. Discussions

### 4.1. LEARNS Mode Could Effectively Reduce the Perioperative Anxiety Level of Patients with Laryngeal Tumors

The results of this study showed that the anxiety level of the two groups of patients with laryngeal tumors was reduced after operation. In the observation group with the LEARNS model implementation, the anxiety score decreased from 61.50 ± 10.33 (1 day before surgery) to 55.23 ± 9.96 (7 days after surgery), and the perioperative anxiety level was significantly lower as compared with the control group with conventional health education model. This indicates that conventional health education is an effective health education model that is widely used and verified by clinical practice to relieve the stress and anxiety of perioperative patients [15–17]. However, the conventional health education model in health care focuses on the evaluation, diagnosis, treatment and nursing of the clinical symptoms or positive indicators of disease progression [18]. According to a previous study, most of nurses in clinical cares have insufficient empathy and motivation to assess the health needs and feelings of the patients [19]. Additionally, routine patient education by nurses usually happens in one-way guidance and passive reception manner, which does not foster self-evaluation and autonomous learning by the patients [20].

In contrast, LEARNS model is an evidence-based practical education process of two-way learning between nurses and patients. It follows the patient-centered principle, which prompts nurses to engage both their mind and body in understanding the needs and feeling of the patients [21]. On the other hand, the involvement of patients in the design of learning program and needs assessment can also motivate the independent learning and promote the health literacy of the patients. The LEARNS model therefore not only promotes the interactive therapeutic partnership, but also fosters evidence-based patient education and learning processes. As sufficient interactions with patients and evidence-based education have been demonstrated to improve the treatment outcome [22], LEARNS model which requires nurses to actively converse with patients instead of waiting for their complaints are expected to ameliorate the anxiety of the patients. This is particularly important for patients with laryngectomy, who are prone to a series of mental problems such as communication disorders, decline in social function, and self-esteem decline after surgery [23, 24].

### 4.2. LEARNS Model Improves the Perioperative Treatment Compliance and Nursing Satisfaction of Patients with Laryngeal Tumors

In this study, the treatment compliance of the two groups of patients was evaluated on the 7th day after the operation, and the nursing satisfaction was evaluated on the day of discharge. The results showed that the perioperative treatment compliance and nursing satisfaction of LEARNS model group was significantly higher than the group receiving conventional health education. The majority of patients with laryngeal tumors were diagnosed and treated in the middle and late stages of the tumor. Although they are treated with total or partial laryngectomy, the generally poor prognosis and the long course of the disease progression makes the expectation of recovery very low [25]. Conventional health education mainly focuses on treatment and education for specific clinical symptoms. The lack of patient needs and feeling assessment makes it difficult to meet the overall psychological, spiritual, social, and cultural needs of patients, which may lower the treatment compliance [26].

The LEARNS model based on the best practice guidelines not only embodies humanistic care, but also implicates an active and interactive patient education program for patients with laryngeal tumors. A previous study indicates that, the awareness of the disease and acceptance of the prognosis are key factors affecting the therapeutic compliance [27]. The response of a patient to treatment is also closely related to their cognitive evaluation and emotional experience of the disease [28]. Therefore, positive cognitive evaluation and emotion regulation can improve health promotion behavior and the acceptance of the treatment. In this study, the LEARNS model was used to fully assess the knowledge of patients with laryngeal tumors on the outcome and development of their own diseases, evaluated the health needs of patients, and establish cooperative partnerships between nurses and patients. The patients were also involved in the formulation and implementation of health education programs to reinforce their health literacy, which could contribute to the improved compliance with perioperative treatment.

### 4.3. LEARNS Model Improves the Perioperative Self-Care Ability and Quality of Life of Patients with Laryngeal Tumors

In this study, the self-care ability of patients was evaluated at 4 weeks after operation, and the quality of life was evaluated at 12 weeks after operation. The results showed that the self-care ability and life quality in the observation group were higher than those of the control group (*P* < 0.05). The score of self-care skills, self-responsibility, self-concept and health knowledge level were also higher than those of the control group, and the health knowledge level showing the most obvious advantage. This indicates that the LEARNS health education model is more organized, structured and purposeful. It is based on evidence-based practice and combined with the preliminary evaluation results to clarify the gap of self-care skills and knowledge (including tracheal tube replacement, cleaning, disinfection, gas Tract humidification, tracheotomy wound care, treatment of tracheal tube blockage, tube slippage, wound bleeding and functional exercises, etc.). Through the cooperation with patients and multi-professional teams, targeted health education programs could improve self-care ability and skills more efficiently.

In addition, after the application of LEARNS model, the sense of self-responsibility and self-awareness have been significantly improved. This may be attributable to the teaching feedback evaluation [29]. Nurses actively conducted dialogues with patients through written materials, telephone calls, computer technology and multimedia. This could strengthen the self-management skills and foster continuous learning.

Our study suffers from the following limitations: (1) The sample size is small and increased cohort number from multiple clinical centers would provide more solid conclusions; (2) During the analysis and patient recruitment, other confounding factors such as family history of exercise and education, and the accessibility to the community health care resources need to be reconciled; (3) A prolonged duration of follow-up would provide more insights into whether LEARNS module procures beneficial effect on the prognosis of the patients.

## 5. Conclusion

The LEARNS model based on the best practice guidelines is a new form of perioperative health education for patients with laryngeal tumors. The core guideline is to create a client-centered care environment without embarrassment and blame, which relieves patient anxiety and effectively implement high-quality nursing. The therapeutic partnership with patients not only helps build knowledge and skills essential for postoperative self-management, but also improves the satisfaction of both nurses and patients. Evidence-based practical education also contributes to enhanced self-care ability, improved therapeutic compliance and an overall higher quality of life.

## Figures and Tables

**Table 1:** Analysis of the gap between LEARNS guidelines and current health education.

Guide mode	Guide meaning	Current clinical status	Ways to improve
L (listen)	Understand patient needs	Based on symptoms and positive indicators	Conduct a needs assessment
E (establish)	Establish a therapeutic partnership	Preaching teacher-student relationship	Master guidelines and standardized training, change nursing concept
A (adopt)	Adopt intentional intervention	Indoctrination	Evaluate the individuality and differential needs, plan multi-mode education
R (reinforce)	Improve health awareness	Based mainly on biochemical indicators	Focus on the psychology and improve self-management efficiency
N (name)	Learning feedback	No systematic evaluation of the educational process	Develop a health education process and dynamically evaluate the outcome
S (strengthen)	Self-management using community resources	Short-term education during hospitalization	Continue nursing health education with the assistance of information platform

**Table 2:** Assessment of learning and needs of patients with laryngeal tumors.

Survey item	Demand content (in order of priority)
Learning duration ways of learning	Study 1 hour a day or study every other day oral explanations and demonstrations given by nurses, video materials playing, official guidelines from the hospital, information searching on internet
Learning tools	Video, audio, pictures, leaflets, brochures, science and education books, web materials
Learning contents	Cannula disinfection, wound dressing, airway humidification, postoperative nutritional management, handling of common accidents in the cannula, learning platform use, psychological adjustment, guidance on pronunciation and communication methods, review content and procedures
Resources	Internet search, science books, hospital information platform, professional books, community presentations

**Table 3:** Comparison of anxiety levels between the two groups of patients (*n*, *X* ± *S*).

	Number (*n*)	Anxiety score on admission	Anxiety score 1 day before surgery	Anxiety score 7 days after surgery
Observation group	30	57.33 ± 12.63	61.50 ± 10.33	55.23 ± 9.96
Control group	30	57.23 ± 13.39	66.83 ± 9.99	60.57 ± 9.87
*t* value		0.030	−2.033	−2.084
*P* value		0.976	0.047	0.042

Statistics: unpaired student's *t* test.

**Table 4:** Comparison of treatment compliance and nursing satisfaction.

	Number (*n*)	Treatment compliance (*n*)			Nursing satisfaction (*n*)		
		*A*	*B*	*C*	Very satisfied	Relatively satisfied	Not satisfied
Observation group	30	21	8	1	20	9	1
Control group	30	13	13	4	12	15	3
*Z* value		−2.175			−2.104		
*P* value		<0.05			<0.05		

Statistics: rank sum test.

**Table 5:** Comparison of postoperative self-care ability and quality of life between the two groups (*n*, %, *X* ± *S*).

	Number (*n*)	Self-care ability score				Life quality score
		Low	Medium	High	Total score (*X* ± *S*)	
Observation group	30	0	1 (3.33%)	29 (96.67%)	131.23 ± 11.21	85.23 + 8.74
Control group	30	0	7 (23.33%)	23 (76.67%)	125.10 ± 10.67	80.03 + 9.68
*X * ^2^ * /t* value		5.192			2.170	2.182
*P* value		0.023			0.034	0.033

Statistics: chi-square (*χ*^2^) statistic test.

## Data Availability

All data used in this study are presented in the manuscript.

## Supplementary Materials

Supplementary Materials Categories of questions in Self-Rating Anxiety Scale (SAS) and Exercise Self-care Agency (ESCA) assessment.
