# Supplementary material for: LEARNS Model as Perioperative Education Strategy for Patients with Laryngeal Tumors
Source: Evid Based Complement Alternat Med. 2022 Oct 12;2022:3360966. doi: 10.1155/2022/3360966 (PMC9581709; doi:10.1155/2022/3360966)
Supplement: Supplementary Materials — Categories of questions in Self-Rating Anxiety Scale (SAS) and Exercise Self-care Agency (ESCA) assessment. [file 3360966.f1.docx]

**Supplementary information:**

**Self-Rating Anxiety Scale，SAS——20**

**1. I feel more nervous or anxious than usual***

**2. I feel afraid for no reason***

**3..I'm easily upset or frightened***

**4. I think I might go crazy***

**5. I think everything will be fine and nothing will happen***

**6. My hands and feet tremble***

**7. I suffer from headache, neck pain and back pain***

**8. I feel weak and tired easily***

**9. I feel calm and easy to sit quietly***

**10. My heart beats fast***

**11. I was troubled by bouts of dizziness***

**12. I have fainted or feel like fainting***

**13. It's easy for me to breathe in and out***

**14. My hands and feet are numb and tingling***

**15. I'm suffering from stomachache and indigestion***

**16. I often have to urinate***

**17. My hands are often dry and warm***

**18. My face is red and hot***

**19. I fall asleep easily and have a good night's sleep***

**20. I have nightmares***

**Exercise Self-care Agency，ESCA**

**self-care skills--12**

**1. If it can improve my health, I am willing to give up some fixed living habits**

**2. I like myself**

**3. I often feel unable to take care of my health in the way I want**

**4. When my health deteriorates, I will find some common sense to improve my health**

**5. I am proud of what I have done to maintain my health**

**6. I often neglect my own needs**

**7. I know my strengths and weaknesses**

**8. When I can't take care of myself, I ask for help**

**9. I will make some new plans (such as those related to health)**

**10. I often give up things that are good for my health**

**11. I will use some effective family secrets without asking medical staff for help**

**12. I can make my own decisions (e.g. health related)**

**self-responsibility--8**

**13. I will do some activities to avoid getting sick**

**14. I try to make myself better (e.g. healthy)**

**15. I eat a balanced diet**

**16. I complain about things that bother me, but I seldom take action to improve it**

**17. I will find some ways to take care of my health**

**18. I hope to attain the best health**

**19. When I have a problem, I need an expert to tell me what to do**

**20. In order to maintain my health, the time spent taking care of myself is valuable**

**self-management literacy--9**

**21. I will carry out the decisions I make**

**22. I am not interested in learning about my own physical functions**

**23. If I'm not good to myself, I can't be good to others**

**24. I understand my body function**

**25. I seldom carry out health-related activities**

**26. I am my good friend**

**27. I take good care of myself**

**28. I occasionally think of promoting my health**

**29. I will arrange my rest and exercise time**

**health knowledge level—14**

**30. I am interested in understanding the process of various diseases and their impact on me**

**31. I think life is a happy thing**

**32. I feel that I have made little contribution to the operation of my family**

**33. I will be responsible for my actions**

**34. I have made little contribution to others**

**35. A few days before I got sick, I would know in advance where I might have a problem**

**36. After years of experience, I already know what to do to make myself comfortable**

**37. I know what to eat to maintain my health**

**38. I am interested in understanding my own body and its functions**

**39. Sometimes, when I feel sick, I will overlook this feeling and hope it will disappear**

**40. I will find some relevant knowledge to take care of myself**

**41. I feel that I am an important member of my family**

**42. I remember the time of the last health check-up, and I will do it next time**

**43. I understand myself and my needs**
